# Supplementary material for: Predictive values of inflammatory back pain, positive HLA B27 antigen and acute and chronic magnetic resonance changes in early diagnosis of Spondyloarthritis. A study of 133 patients
Source: PLoS One. 2020 Dec 21;15(12):e0244184. doi: 10.1371/journal.pone.0244184 (PMC7751977; doi:10.1371/journal.pone.0244184)
Supplement: S1 Table — (DOCX) [file pone.0244184.s001.DOCX]

**S1 Table**

MRI parameters.

|  | MRI PARAMETERS | | | | | | | |
| --- | --- | --- | --- | --- | --- | --- | --- | --- |
| Sequences | Slices | Slice thickness  (mm) | Interslice gap  (mm) | Field of view  (mm) | Repetition time  (msec) | Echo time  (msec) | Turbo factor | Matrix  (pixels) |
| Coronal Tw1 | 25 | 3.5 | 0.7 | 320 | 886 | 11 | 2 | 240x320 |
| Axial Tw1 | 25 | 4 | 1.4 | 320 | 485 | 12 | 2 | 240x320 |
| Coronal STIR | 25 | 4 | 0.4 | 380 | 3630 | 31 | 8 | 113x256 |
| Axial STIR | 25 | 4 | 0.4 | 320 | 3670 | 32 | 9 | 192x256 |
| Coronal Tw2 | 25 | 4 | 0.4 | 380 | 5580 | 89 | 17 | 171x384 |

Tw1- T1-weighted sequence, STIR -­ short tau inversion recovery sequence, Tw2-T2-weighted sequence.
