# Supplementary material for: Predictive values of inflammatory back pain, positive HLA B27 antigen and acute and chronic magnetic resonance changes in early diagnosis of Spondyloarthritis. A study of 133 patients
Source: PLoS One. 2020 Dec 21;15(12):e0244184. doi: 10.1371/journal.pone.0244184 (PMC7751977; doi:10.1371/journal.pone.0244184)
Supplement: S2 Table — (DOCX) [file pone.0244184.s002.DOCX]

**S2 Table**

Statistical analysis.

* Chi-square test, ** T Student test, *** Mann-Whitney U test, **** Fisher's exact test
