# Supplementary material for: Predictive values of inflammatory back pain, positive HLA B27 antigen and acute and chronic magnetic resonance changes in early diagnosis of Spondyloarthritis. A study of 133 patients
Source: PLoS One. 2020 Dec 21;15(12):e0244184. doi: 10.1371/journal.pone.0244184 (PMC7751977; doi:10.1371/journal.pone.0244184)
Supplement: S3 Table — (DOCX) [file pone.0244184.s003.DOCX]

**S3 Table**

Predictive values.

PPV – predictive positive value, PNV- predictive negative value,

CI- confidence interval.
