# Supplementary material for: Predictive values of inflammatory back pain, positive HLA B27 antigen and acute and chronic magnetic resonance changes in early diagnosis of Spondyloarthritis. A study of 133 patients
Source: PLoS One. 2020 Dec 21;15(12):e0244184. doi: 10.1371/journal.pone.0244184 (PMC7751977; doi:10.1371/journal.pone.0244184)
Supplement: S4 Table — (DOCX) [file pone.0244184.s004.DOCX]

**S4 Table 4**

Multivariate logistic regression binary analysis.

*IBP- inflammatory back pain, **CRP – C reactive protein, ***SpAF - SpA features,

OR-Odds Ratio, AUC- area under the curve, SE- standard error.
