# Supplementary material for: Predictive values of inflammatory back pain, positive HLA B27 antigen and acute and chronic magnetic resonance changes in early diagnosis of Spondyloarthritis. A study of 133 patients
Source: PLoS One. 2020 Dec 21;15(12):e0244184. doi: 10.1371/journal.pone.0244184 (PMC7751977; doi:10.1371/journal.pone.0244184)
Supplement: S5 Table — Study comparison. (DOCX) [file pone.0244184.s005.DOCX]

**S5 Table**

Tw1 and STIR MRI imaging reliability. Study comparison.

|  | **MRI evaluation** | | **Lesions number** | **Sensitivity** | **Specificity** | **Inter-observer agreement** |
| --- | --- | --- | --- | --- | --- | --- |
| **Aydin et al. (19)** | Global assessment*  ASAS** | | BME ≥2  BME ≥2 | 0.66  0.79 | 0.94  0.89 | Any two readers |
| **Joven et al. (20)** | ASAS** | | BME ≥2 | 0.48 | 0.92 | NR^ |
| **Weber et al. (23)** | Global assessment *  Confidence level***  ASAS**  SPARCC+  Detailed scoring of MRI lesions | MRI criterion as gold standard | BME ≥2  Erosion ≥1  BME ≥2 and/or erosion ≥1 | 0.90  0.90  0.90 | 0.91  1  0.98 | 0.76/0.80 (total group) |
|  |  | Clinical classification as gold standard | BME ≥3  Erosion ≥1  BME ≥2 and/or erosion ≥1 | 0.73  0.77  0.82 | 0.90  0.90  0.90 |  |
| **Weber et al. (24)** | Global assessment *  ASAS**  SPARCC+  Detailed scoring of MRI lesions | | BME ≥2  Erosion s≥2  BME ≥2 and/or erosion ≥1  BME ≥3 and/or erosions ≥2 | 0.80  0.58  0.88  0.83 | 0.76  0.97  0.72  0.85 | 0.88/0.75  0.82/0.71  NR^  NR^ |
| **Oliveira et al. (25)** | Global assessment *  Confidence level***  ASAS**  SPARCC+  Detailed scoring of MRI lesions | | BME ≥2  BME ≥3  Erosions ≥2  BME ≥2 and/or erosion ≥2  Fat metaplasia ≥2  Backfill ≥2 | 0.94  0.88  0.47  1  0.35  0.29 | 0.83  0.94  0.96  0.80  0.98  1 | 0.54  NR^  0.68  NR^  0.72  0.70 |
| **Molto et al. (27)** | Global assessment *  ASAS**  SPARCC+  Detailed scoring of MRI lesions | | Any structural lesion ≥1  BME ≥3  Fat metaplasia ≥3  Erosion or fat metaplasia ≥5 | 0.20  0.30  0.30  0.30 | 0.80  0.90  0.90  0.90 | NR^ |
| **Our study** | Global assessment*  Confidence level++  ASAS**  Detailed scoring of MRI lesions | | SPBME E≥2  WPBME ≥2  Erosion ≥1  SPBME ≥2 and erosion ≥1  WPBME ≥2 and erosion ≥1  Fat metaplasia ≥1  Backfill ≥1 | 0.79  0.87  0.72  0.62  0.64  0.57  0.39 | 0.94  0.66  0.93  1  0.98  0.83  1 | 0.76  0.59  0.47  NR^  NR^  0.44  0.51 |

*Global assessment (T1 and STIR simultaneously), presence or absence of SpA.

**ASAS- Assessment of SpondyloArthritis international Society definition.

***Confidence level of readers with global assessment classification.

+SPARCC- Spondyloarthritis Research Consortium of Canada MRI index for SIJ.

++Confidence level of readers with ASAS definition.

^NR – not reported.
