# Supplementary material for: Predictive values of inflammatory back pain, positive HLA B27 antigen and acute and chronic magnetic resonance changes in early diagnosis of Spondyloarthritis. A study of 133 patients
Source: PLoS One. 2020 Dec 21;15(12):e0244184. doi: 10.1371/journal.pone.0244184 (PMC7751977; doi:10.1371/journal.pone.0244184)
Supplement: Attachment — Submitted filename: Answers to Reviewer #2 (1).docx [file pone.0244184.s006.docx]

Reviewer #2: This study aims to assess the impact of MRI lesions in the sacroiliac joint on the early diagnosis of axSpA. It is concluded that the addition of erosion to BME detected by MRI enhances specificity for diagnosis without significantly compromising sensitivity. I have several suggestions to improve the quality of the manuscript.

*Dear Reviewer,*

*We are incredibly grateful for your comments. Bearing in mind your suggestions we tried to improve the quality of our work and address your points of concern. Please, see the answers below.*

1. There is no relevant introduction to this manuscript. The introduction should include a synopsis of the scientific question and/or unmet need followed by a statement of study hypothesis and/or objectives. This needs to be re-written.

*Answer:* We agree with your suggestions. The introduction has been entirely re-written. You can see changes on page 2, lines 48 to 89.

2. Methods: “To define STIR acute inflammatory lesions (BME) we used the ASAS/ OMERACT definition, accepting for positive MRI the presence of BME ≥2, depicted as a strong hyperintense signal similar to that of the blood vessels”. What does the score of BME≥2 mean and what is the origin of this score?

*Answer:* Thank you again for your time. We have now explained the origin and the meaning of the score of BME≥2. Please, see our comments on page 3, section MRI evaluation, lines 129-140.

3. Methods: “We divided STIR sequences in three categories, strongly positive (SP) BME ≥2, if images fulfilled ASAS/OMERACT criterion and they were clearly presented and easily recognizable as positive by readers (high level of confidence), and weakly positive (WP) BME ≥2, if lesions fulfilled ASAS/OMERACT criterion, but they were tenuous and not easily identifiable as positive (low level of confidence).”
The rationale for using this method should be supported by a citation. Many would argue that this is a flawed method because previous work has shown that up to 40% of healthy individuals will have BME meeting the ASAS-OMERACT criterion.

*Answer:* Thank you for pointing it out. The reason for using a semi-quantitative way to define STIR imaging instead of the quantitative SPARACC scoring model was an attempt to better reflect a circumstance mentioned by the ASAS guidelines for the application of a definition of a positive MRI (active sacroiliitis), where BME appeared to be present but it was hard to determine if the ASAS criteria “highly suggestive” was met. In those cases, the decision might be influenced by the presence of concomitant structural lesions You can see changes on page 3 lines 140-141 and page 4 lines 142 -145, also abstract page 1, lines 40 -42.

4. Methods: “Sacroiliac radiographies were evaluated by the rheumatologist and assessed according to The Modified New York criteria (9). Radiographies were graded as positive if there were at least bilateral grade 2, or unilateral grade 3 sacroiliitis.”
The radiographs should have been assessed by 2 readers plus adjudicator just like the MRI scans.

*Answer:* We partially disagree with the expert opinion. We understand that for a greater scientific contribution at least 3 readers are necessary, however radiographic outcomes have not been a primary nor a secondary aim in our study. To avoid confusion, we removed all data where multivariate analysis were performed for patients with nr-axilSpA and exposed only the data that referred to the entire group of patients

5. The statistics section does not indicate what is the primary outcome, specifically how the final diagnosis was ascertained.

*Answer:* Thank you for focussing on this issue. We have added more data.

All patients were evaluated during the follow-up period by the rheumatologist and final diagnosis was based on his clinical opinion, settled on clinical examination, laboratory values and imaging (MRI and radiographies). Where possible, Modified New- York criterion was applied. The authors realized that in patients with pre-radiographic sacroiliitis diagnosis was influenced by the ASAS group classification criteria, nonetheless the clinical opinion was mandatory for the final diagnosis and this used as a gold standard for the study purpose. Please see changes on page 3, section Clinical evaluation, lines 103-109.

All end-point variables were tested against the gold standard (i.e the rheumatologist´s diagnosis at the end of the follow-up period). Please see changes on also page 4, section Statistical analysis, lines 166-168.

6. Results: “The average age in our study was 38.9 years with male/female ratio 0.41/1. 47 (35.3%) patients received diagnosis of SpA. 8 (17%) patients had radiographic sacroiliitis. IBP was found in 55 (41.4%) patients, positive HLA B 27 antigen in 40 (30.3%), increased CRP in 25 (18.8%) and SpAF in 25 (18.8%).”
SpA is typically a male disease with B27 prevalence >80%. In the SpA patients B27 positivity was only 50% casting doubt on the diagnostic process.

*Answer:* We disagree with the expert opinion. Recent studies have demonstrated differences between men and women with recent-onset axial SpA and longer delay in diagnosis in women compared to men. You can see all our arguments on page 8, Discussion section, lines 337-360.

7. Results: “SP BME ≥2 (78.7% vs 6%), sensitivity, 0.79, specificity 0.94 (p<0.001), as well as, WP BME ≥2 (87% vs 33.7%), sensitivity 0.87, specificity, 0.66 (p<0.001).”
It should be stated how reliably SP versus WP was detected.

*Answer:* Definition for SPBME≥2 and WPBME≥2 was previously explained. As we have said this is a semi-qualitative way to define the confidence level when readers are scoring STIR images. We considered to use this way instead of using the quantitative SPARCC scoring model in an attempt to better approach a real clinical situation. The term SPBME≥2 reflects a high reader´s confidence level (6-10) when evaluating STIR image as positive according to the ASAS criterion, while WPBME≥2 reflects lower confidence level (0-4). MRI images with confidence level 5 were re-evaluated until one of the two previously mentioned confidence levels was achieved. See more method section, subparagraph “To define active sacroiliitis on STIR sequences”, page 3, lines 129-141, and page 4- lines 142-145.

8. Results: “Those patients had also more often erosion ≥1 (72% vs 7%), (p<0.001).”
Which patients does this refer to,….the SP BME?

*Answer:* Thank you very much for pointing this out. The section MRI findings has been re- written, and we have tried to better explain all outcomes. You can see our comments on page 5, lines 199-219.

9. Results: “ROC curve, plotting true positive rate against false positive rate, with area under the curve (AUC) at least 50% was calculated, showing AUC, 91.6%, for patients with nr-axSpA, and 91.4%, for the total group (p<0.001).”
It should be clarified what variable is being analyzed in the AUC.

*Answer:* We agree with your suggestion. The section Multivariate analysis has been re- written, and we have tried to better explain all details. You can see our comments on page 5, lines 220-231.

10. Results: “IOA between readers for T1wCL was as follows; erosion 0.47 (0.35; 0.59), backfill 0.51 (0.35; 0.66), fat metaplasia 0.44 (0.32; 0.56), and sclerosis 0.53 (0.41; 0.64) (p<0.001) (see table 5).”
This does not appear to be sufficiently reliable. It should be stated what training or calibration was undertaken by the readers.

*Answer:* We disagree with the expert opinion. We have explained our point of view. Regarding the first question please, see our arguments on page 7, lines 319-327.

Regarding the second point we added that “Readers were previously calibrated with a training session and prereading exercises were developed, setting the threshold for detection of the acute and chronic MRI lesions.” you can see new data on page 4, lines 159-161.
